# Supplementary material for: Boundary curvature guided programmable shape-morphing kirigami sheets
Source: Nat Commun. 2022 Jan 26;13:530. doi: 10.1038/s41467-022-28187-x (PMC8792031; doi:10.1038/s41467-022-28187-x)
Supplement: Supplementary file 1 — Supplementary information [file 41467_2022_28187_MOESM1_ESM.pdf]

Supplementary Information for

Boundary curvature guided programmable shape-morphing kirigami sheets

Yaoye Hong[1], Yinding Chi[1], Shuang Wu[1], Yanbin Li[1], Yong Zhu[1], Jie Yin[1,*]

[1]Department of Mechanical and Aerospace Engineering, North Carolina State University, Raleigh, NC 27695, USA.

[*]To whom correspondence may be addressed. Email: jyin8@ncsu.edu

Supplementary note 1. Theoretical model of the three characteristic shapes

Supplementary note 2. Gaussian curvature dependence on the boundary curvature

Supplementary note 3. Effect of the boundary curve smoothness in combinatorial designs

Supplementary note 4. Actuated ribbon bistability by remote magnetic field

Supplementary note 5. Inverse design

Supplementary note 6. Performance of the modified kirigami grippers

Supplementary note 7. Maximum tensile strain in the ribbons

Supplementary note 8. Extending the strategy to other cut patterns

Supplementary Figs. 1 - 17

Legends for supplementary Movie 1-3

Supplementary note 1. Theoretical model of the three characteristic shapes

Deformation of the discrete ribbons. The torsional energy of the boundary ribbon is small compared with the bending energy of discrete ribbons due to the small width of the boundary ribbon. Therefore, the torsion of the boundary ribbon is dominated by discrete ribbons. As such, the shape of the slender discrete ribbons can be derived based on large deflections of buckled ribbons[1] and is expressed as

$$\bar{y}_d = \frac{2}{\lambda} E(AM(\lambda \bar{s}_d, m), m) - \bar{s}_d \qquad [1a]$$

$$\bar{z}_d = \frac{2m}{\lambda} CN(\lambda \bar{s}_d, m) \qquad [1b]$$

where $\bar{y}_d$ and $\bar{z}_d$ are normalized coordinate functions; $\bar{s}_d$ denotes the normalized arc length coordinate of the discrete ribbons; the discrete ribbon is parametrized by its arc length and these variables are normalized with the half-width of the precursor, *R*; the origins are located at the midpoint of ribbons as shown in Supplementary Fig. 2； *E*, *AM*, and *CN* are elliptic functions; $m(\bar{s}_b, \varepsilon)$ is the modulus characterizing the deformation of the discrete ribbons and can be calculated using Eq. (6) in the main text. The angle φ between the plane T_b containing the boundary ribbon and the normal plane T_n of the discrete ribbon at this point (Supplementary Fig. 3) is dependent on m and is expressed as

$$\varphi = \tan^{-1}\left(-\frac{2\sqrt{m^2 - m^4}}{1 - 2m^2}\right) + \frac{\pi}{2} \qquad [2]$$

Deformation of the boundary ribbons. The applied strain of the three characteristic shapes (Supplementary Fig. 2) is given by

$$\varepsilon = \frac{\bar{l} - \bar{l}_0}{\bar{l}_0} \qquad [3]$$

where $\bar{l}_0 = 2$ and $\bar{l}$ are the normalized lengths of the shapes by the half-width of the precursor, R, before and during deformation, respectively.

First, for the cylindrical shape, it is obvious that $\bar{s}_b, \bar{s}_d \in [-1,1]$, where $\bar{s}_b = \frac{s_b}{R}, \bar{s}_d = \frac{s_d}{R}$. The explicit expression of the boundary ribbon is in the form of

$$f(\overline{s}_b, \varepsilon) = \overline{s}_b \quad [4a]$$

$$g(\overline{s}_b, \varepsilon) = \sqrt{1 - \varepsilon^2} \quad [4b]$$

where $(f(\overline{s}_b, \varepsilon), g(\overline{s}_b, \varepsilon), 0)$ denotes the normalized Cartesian coordinates of the boundary ribbon. As shown in Supplementary Fig. 2, the strain ε is expressed as $\varepsilon = \frac{\bar{l} - \bar{l}_0}{\bar{l}_0} = \frac{\sqrt{(l_s)^2 - (l_w/2)^2}}{R} = \sqrt{1 - g^2}$, where l_s and l_w are the length of the stretching ribbon and the width of the shape, respectively; the maximum strain is given by $\max(\varepsilon)$ $\varepsilon\_max = \frac{2 l_s}{l_0} \approx 1$.

Also, the rotation of the boundary ribbon does not change the plane containing the discrete ribbon. The tilting angle α_1 between the discrete ribbon and the xy plane keeps unchanged in the cylindrical case (Supplementary Fig. 4) and is given by

$$\alpha_1 = \alpha_{2s} = \alpha_2 = \frac{\pi}{2} \quad [5]$$

Second, for the spheroidal structure, it is obvious $\overline{s}_b \in \left[-\frac{\pi}{2}, \frac{\pi}{2}\right]$ and $\overline{s}_d \in [-\cos \overline{s}_b, \cos \overline{s}_b]$. The shape of the boundary ribbon in the xy plane is expressed as

$$f(\overline{s}_b, \varepsilon) = (1 - \overline{w}) \sin \theta + \overline{v} \cos \theta \quad [6a]$$

$$g(\overline{s}_b, \varepsilon) = (1 - \overline{w}) \cos \theta - \overline{v} \sin \theta \quad [6b]$$

where $(f(\overline{s}_b, \varepsilon), g(\overline{s}_b, \varepsilon), 0)$ denotes the normalized Cartesian coordinates at $\overline{s}_b$ of the boundary ribbon. $\overline{w}(\overline{s}_b, \varepsilon)$ and $\overline{v}(\overline{s}_b, \varepsilon)$ denote the normalized radial and tangential displacement of the boundary ribbon, respectively. θ is expressed as $\theta = \overline{s}_b$ and there exists a mismatching between θ and $\overline{s}_b$ at a large strain, which needs to be modified using the arclength, or it will cause an error of 6% near the end of the boundary curve. The applied strain ε is expressed as $\varepsilon = \frac{\bar{l} - \bar{l}_0}{\bar{l}_0} = f(\frac{\pi}{2}, \varepsilon) - 1$ and the maximum strain is given by $\max(\varepsilon) \varepsilon_{max} = \frac{\pi - 2}{2} \approx 0.57$.

The boundary ring is under the effect of both the applied tensile force P and the reaction force generated by buckled discrete ribbons. Because the moment produced by reaction force in the boundary ring is small compared with that generated by critical buckling pressure of circular rings[2],

we assume that the effect of the reaction force is equivalent to that of the equivalent force P_e along the y axis at $\overline{s}_b = 0$. Also, we assume that P_e linearly increases with increasing P based on the relationship among P_e, P, and the radial displacement. Therefore, the normalized tangential and radial displacement are in the form of $\overline{w} = -c\{\left(\cos\theta + \theta\sin\theta - \frac{4}{\pi}\right) + r_b\left(\cos(\frac{\pi}{2} - \theta) + (\frac{\pi}{2} - \theta)\sin(\frac{\pi}{2} - \theta) - \frac{4}{\pi}\right)\}$, $\overline{v} = -c\left\{\left(2\sin\theta - \theta\cos\theta - \frac{4\theta}{\pi}\right) + r_b\left(2\sin\left(\frac{\pi}{2} - \theta\right) - \left(\frac{\pi}{2} - \theta\right)\cos\left(\frac{\pi}{2} - \theta\right) - \frac{4\left(\frac{\pi}{2} - \theta\right)}{\pi}\right)\right\}$, where $r_b = \frac{\pi N w_d t^2}{4{w_b}^3}$ is the ratio of the equivalent force generated by buckled discrete ribbons and the applied stretching force. w_d, w_b, and t denote the width of discrete ribbons, the width of boundary ribbons, and the thickness of the ribbons. N is half the number of discrete ribbons due to the symmetry of structures. c is a dimensionless variable related to the magnitude of the normalized tensile force. Additionally, the model uses the linear inextensibility condition and neglects higher-order terms, which needs to be modified using high order terms under large strain.

It is also worth noting that the morphology is independent of the width of the discrete ribbon if $w_d \ll l_0$. The width of the cuts is neglected， and Nw_d does not change， leading to the constant r_b. Moreover, during deformation, the angle $\alpha_{2s} = \alpha_2$ does not change due to the conformal mapping. As shown in Supplementary Fig. 4, it is obvious $|AO|^2 + |MO|^2 - |AM|^2 = 2|AO||MO|\cos\alpha_1$. Accordingly, the angle α_1 is expressed as

$$\alpha_1(\overline{s}_b, m) = \cos^{-1}\{\tan(\varphi)\left[\frac{1}{2\tan(\theta_1)} - \frac{\tan(\theta_1)}{2} - \frac{1}{\sin(2\theta_1)} + \frac{\cos(\alpha_{2s})}{\sin(\varphi)\cos(\theta_1)}\right]\} \quad [7]$$

where $\alpha_{2s} = \alpha_2$ is the angle between the tangent line of the boundary ribbon and the tangent line of the discrete ribbon (Supplementary Fig. 4). θ_1 denotes the angle between the tangent line of the boundary ribbon and the x axis. θ_1 can be simplified as $\theta_1 = \overline{s}_b$ at a small strain and the general equation of θ_1 is expressed as $\theta_1 = \frac{dg(\overline{s}_b,\varepsilon)}{df(\overline{s}_b,\varepsilon)}$.

Third, for the saddle shape, the domain of the explicit expression is given by $\overline{s}_b \in [-1.32, 1.32]$ and $\overline{s}_d \in [\cos\overline{s}_b - 1.5, 1.5 - \cos\overline{s}_b]$; note that the normalized maximum boundary arc length is smaller than $\frac{\pi}{2}$ and the actual limit of $\overline{s}_d$ is smaller due to the width of the stretching

ribbon (Supplementary Fig. 2). After the critical strain ($\varepsilon_c = 1.42$), the boundary ribbon keeps a straight line and the shape of the boundary ribbon is expressed as

$$f(\bar{s}_b, \varepsilon) = \bar{s}_b \quad [8a]$$

$$g(\bar{s}_b, \varepsilon) = \sqrt{(\bar{l}_s)^2 - (\varepsilon - 0.32)^2} \quad [8b]$$

where $(f(\bar{s}_b, \varepsilon), g(\bar{s}_b, \varepsilon), 0)$ denotes the normalized Cartesian coordinates of the boundary ribbon; $\bar{l}_s$ is the normalized length of the stretching ribbon; the strain ε is given by $\varepsilon = \frac{\sqrt{l_s{}^2-(l_w/2)^2} + \left(\frac{\pi}{2}-0.25\right)R-R}{R} = \sqrt{\bar{l}_s{}^2 - g^2} + 0.32$ as shown in Supplementary Fig. 2; the maximum strain is given by $\max(\varepsilon) = \bar{l}_s + 0.32 \approx 1.53$.

During deformation, the angle α_{2s} changes due to the contact between the discrete ribbons. The tilting angle α_1 exhibits a near-linear dependence on $\bar{s}_b$ and is expressed as

$$\alpha_1(\bar{s}_b, m) = \frac{(\alpha_{2c}+\alpha_2)}{2} \quad [9]$$

where $\alpha_{2c} = \tan^{-1}(\frac{\sin\theta}{1-\cos\theta})$.

Additionally, we note that before the critical strain $\varepsilon_c = 1.42$, the structure shows the sequential deformation (Supplementary Fig. 6); the discrete ribbons buckle from the border to the center sequentially. The physical origin of the sequential buckling can be explained from both the differential geometry and mechanics perspectives. Geometrically, the boundary ribbon is gradually straightened from the border ($\bar{s}_b = 1.32$) to the center ($\bar{s}_b = 0$) accompanied by the sequential variation in the curvature of the boundary ribbon. Also, the straight segment (the border) is tangent to the curved segment (the center) of the boundary ribbon. The flattened regions of the boundary curve induce the corresponding discrete ribbons to pop up. This is consistent with the Gauss-Bonnet theorem, where the variation of the geodesic curvature induces the change in the Gaussian curvature. In contrast, for the spheroidal shape, the curvature variation along the circular boundary ribbon happens at the same time. For the cylindrical shape, there is no curvature variation in the boundary ribbon. Mechanically, considering the force equilibrium of the boundary ribbon along the y axis, the sequential deformation is controlled by a dimensionless variable $\delta =$

$\frac{P \sin(2\beta)}{2\sum_{i=1}^{n} P_C^i}$, where P and $\sum_{i=1}^{n} P_C^i$ denote the applied tensile force and the summation of the critical buckling force of the discrete ribbon and $\beta = \sin^{-1}(\frac{g}{\bar{l}_s})$. For a concave boundary, we have $\delta < 1$, leading to a sequential buckling. While for the circular and the rectangular boundaries, we have $\delta = 1$, which means that all the discrete ribbons pop up simultaneously and the sequential buckling behavior ends.

General equation of the morphed shape. Considering the surface foliated by continuously varying discrete ribbons along the boundary ribbon, Eqs. (2-4) in main text are derived by sweeping the varying discrete ribbons (modeled by elastica) along the boundary ribbon. First, given the deformation of the boundary ribbon described above, the deformation of the discrete ribbons characterized by *m* is derived by combining Eq. (6) in main text, supplementary Eq. (4b), Eq. (6b), and Eq. (8b). Then, based on the known explicit expressions of all ribbons, the morphed shape is parameterized by the arc length of the discrete and boundary ribbons. Remarkably, this general equation can be applied to all the morphed shapes in this work, including the tessellated structures (Fig. 4).

Supplementary note 2. Gaussian curvature dependence on the boundary curvature

Principal curvatures at the central point. Based on the validated theoretical model, we further investigate how to manipulate the Gaussian curvature *K* of the morphed topologies by varying the initial boundary curvature k_{bo} of the 2D circular precursors at a given applied strain. For simplification, we focus on the variation of *K* at the representative center point $C(\bar{s}_b = 0, \bar{s}_d = 0)$ (Figs. 3a-b). The normalized Gaussian curvature at this point is expressed as $\bar{K} = \bar{K}(C) = \bar{k}_1\bar{k}_2$, where $\bar{k}_1$ and $\bar{k}_2$ denote the normalized principal curvatures at point *C*. $\bar{k}_1$ lies in the plane formed by **N** and **v₁**; and $\bar{k}_2$ lies in the plane formed by **N** and **v₂** as shown in Supplementary Fig. 8. $\bar{k}_2$ is proportional to the height ($\bar{z}$) of the point and $\bar{k}_1$ is approximated by ellipse curve based on the similarity to demonstrate the global backbone curve instead of the local discontinuity due to the contact between discrete ribbons. Additionally, the principal curvatures are normalized by the half length of the central discrete ribbon. $\bar{k}_1$ and $\bar{k}_2$ are given by

$$\bar{k}_1 = \begin{cases} \dfrac{\bar{z}(0,0)}{\bar{x}^2\left(\gamma/\bar{k}_{bo},0\right)}, & \bar{k}_{bo} > 0 \\ \dfrac{\bar{z}(0,0)-\bar{z}\left(\gamma/\bar{k}_{bo},0\right)}{2\left[\bar{x}\left(\gamma/\bar{k}_{bo},0\right)-\bar{x}(0,0)\right]^2}, & \bar{k}_{bo} < 0 \end{cases} \quad [10a]$$

$$\bar{k}_2 = 2mF\left(\frac{\pi}{2},m\right) \quad [10b]$$

where γ denotes half of the central angle of the boundary curve and $\bar{k}_{bo}$ denotes the normalized initial boundary curvature of the 2D precursor.

Modification due to the variation in the initial boundary curvature. The maximum applied strain $\max(\varepsilon)$ for 2D precursors depends on initial boundary curvature $\bar{k}_{bo}$. We use the normalized applied strain $\bar{\varepsilon}$ to unify precursors with different $\bar{k}_{bo}$, which is expressed as

$$\bar{\varepsilon} = \begin{cases} \frac{\varepsilon}{\max(\varepsilon)}, & \bar{k}_{bo} > 0 \\ \frac{\varepsilon-\varepsilon_C}{\max(\varepsilon)}, & \bar{k}_{bo} < 0 \end{cases} \quad [11]$$

where ε_C is the critical strain of the saddle shape. Note $\bar{K}(C) = 0$ when $\varepsilon < \varepsilon_C$.

The deformation of the structures with various initial boundary curvature can still be modeled using the generalized Eq. (2) in main text, but the θ needs to be modified due to the variation of the initial boundary curvature and the modified θ' is expressed as $\theta' = \pi\theta/(2\gamma)$ according to Ref.[2], where $\theta \in [0,\gamma]$ and $\theta' \in [0,\frac{\pi}{2}]$. γ denotes the half central angle of the boundary curve.

Relationship between the Gaussian curvature and the boundary curvature. For the spheroidal shapes, the normalized variation $\left|\frac{\Delta\bar{K}}{\bar{K}_{max}}\right|$ of the Gaussian curvature is expressed as $\left|\frac{\Delta\bar{K}}{\bar{K}_{max}}\right| = \frac{2mF(\frac{\pi}{2},m)\bar{z}(0,0)}{\bar{K}_{max}\bar{x}^2(\frac{\gamma}{\bar{k}_{bo}},0)}$, where $\bar{z}(0,0) = \frac{2m}{F(\frac{\pi}{2},m)}$. The normalized variation $\left|\frac{\Delta\bar{k}_b}{\bar{k}_{bo}}\right|$ of the boundary curvature is in the form of $\left|\frac{\Delta\bar{k}_b}{\bar{k}_{bo}}\right| = \frac{\bar{k}_{bo}\bar{x}^2(\frac{\gamma}{\bar{k}_{bo}},0)-\bar{y}(0,1)}{\bar{k}_{bo}\bar{x}^2(\frac{\gamma}{\bar{k}_{bo}},0)}$. We use that ellipse curve to approximate the

curves to simplify the calculation based on the similarity. For saddle shapes, we have $\left|\frac{\Delta\bar{K}}{\bar{K}_{max}}\right| / \left|\frac{\Delta\bar{k}_b}{\bar{k}_{b0}}\right| \approx 1$ because of the sudden jump in the Gaussian curvature at the critical strain. For the cylindrical shape, the Gaussian curvature keeps zero, as shown in Fig. 3c. The nonlinearity of the experimental results comes from the change of direction of the geodesic vector.

Analysis of the error induced by the holes. First, $C = 2\pi\chi(\Omega) - \sum_{i=1}^{p}\theta_i$, keeps zero during shape shifting due to the unchanged Euler characteristic and the summation of the exterior angles based on the Gauss-Bonnet theorem[3]. Second, C can also be expressed as $C = \int_{\Omega} KdA + \int_{\partial\Omega} k_{gb}ds$, where $\int_{\Omega} KdA$ and $\int_{\partial\Omega} k_{gb}ds$ are calculated by Eqs. (2)-(4) in main text based on the solid mechanics. To demonstrate the variation of C from these two equations during shape shifting, we use C as a function of the normalized geodesic curvature $\bar{k}_{gb}$ along the boundary ribbon. As shown in Supplementary Fig. 9, $C = \int_{\Omega} KdA + \int_{\partial\Omega} k_{gb}ds$ is slightly larger than $C = 2\pi\chi(\Omega) - \sum_{i=1}^{p}\theta_i$ due to the hollow spaces. However, the discrepancies between the red (from solid mechanics) and orange (from differential geometry) curves are small compared with $\int_{\Omega} KdA$ and can be neglected.

Supplementary note 3. Effect of the boundary curve smoothness in combinatorial designs

Shape dependence on the smoothness of the boundary curve. The smoothness of the backbone in the morphed shape depends on the smoothness of the boundary in the 2D precursor. C^0 continuity represents curves without continuous first derivative; C^1 continuity represents the curve and the first derivative are continuous (i.e., continuous in tangent vector); C^2 continuity represents the curve, the first derivative, and the second derivative are continuous (i.e., continuous in curvature). For instance, the eyes and mouth in the form of holes (Figs. 4a-d) are formed and controlled by its C^0continuous boundaries, arising from the variation of the exterior angles at the joints based on the Gauss-Bonnet theorem. Note that for C^0 continuous boundaries, the decrease of the exterior angles results in a hole, while the increase of the exterior angles leads to the contact of discrete ribbons. The C^2 and C^1 continuous boundary segments generate the sections with C^2

and C^1 continuity in the backbone, respectively, as shown in the waterdrop and vase shape (Figs. 4l, 4n).

Supplementary note 4. Actuated ribbon bistability by remote magnetic field

We note that each ribbon in the buckled shapes of the three kirigami sheets with different boundary curvatures is bistable, i.e., it can pop up or pop down energetically equivalent. By manually flipping the popping directions in the three buckled spheroidal, cylindrical, and saddle shapes under the stretched state, it can transform to more different shapes, as shown in Supplementary Fig. 10 as an example.

In addition to the contact-based mechanical actuation for shape morphing, we also demonstrate the capability of achieving bistability in kirigami sheets through remote magnetic actuation. By attaching magnetic thin polymeric stripes to the discrete ribbons (Supplementary Fig. 11), we could use a remote translational magnetic field to fast switch the bistable states in the ribbons to reconfigure the kirigami structure. Supplementary Fig. 11a shows a sequential snapping of the discrete ribbons in the spheroidal structure, where its sequence could be tuned via the distribution of the magnetic polymers in the 2D kirigami precursor. Supplementary Fig. 11b illustrates the pattern of the magnetic polymer attached to the discrete ribbons. We harness the distribution of the polymers changing the direction and the magnitude of the magnetic forces and torques acting on the discrete ribbons to tune the snapping sequence of the discrete ribbons. It is obvious that with the increasing strength of the magnetic field, ribbon one snaps first and then the ribbon two because the magnetic forces acting on them are larger. The ribbon three snaps last because the magnetic forces acting on it are smaller.

Supplementary note 5. Inverse design strategy

Inverse design of the waterdrop and vase shape. We first use curves to approximate and represent the target shape. Given the shape of the geodesics G and the principal curvatures at the midpoint of discrete ribbons, we use the elastica curves to approximate the geodesics. Based on the generalized equation (Eqs. (2)-(4) in main text) for morphed kirigami structures, the information of one representative geodesic curve is enough for the inverse design. This significantly simplifies the calculation and makes the precise control unnecessary. The

deformation of each geodesic is characterized by the elliptic modulus m; the modulus m for the representative geodesic curve in the waterdrop and vase shape is 0.55 and 0.71, respectively. Accordingly, the target surface is represented by a set of geodesic curves G, two boundary curves Γ, and a backbone curve B.

The parameterization of the backbone curve B is expressed as $\mathbf{r_B} = (x, 0, z)$. The explicit expression of B (waterdrop) is in the form of

$$z = \begin{cases} 0.323x, \ 0 \le x \le 2 \\ -0.326x^7 + 6.79x^6 - 59.9x^5 + 291x^4 - 842x^3 + 1446x^2 - 1365x + 546, 2 < x \le 4.1 \end{cases} \quad [12]$$

where z and x are the Cartesian coordinates of the points in the backbone curve B.

The explicit expression of the backbone curve B (vase) is in the form of

$$z = \begin{cases} 2.32, \ 0 \le x \le 0.43 \\ 0.0944x^3 + 0.628x^2 - 0.0773x + 2.33, \ 0.43 < x \le 1.7 \\ -0.000468x^4 + 0.0138x^3 - 0.214x^2 + 1.68x + 0.561, \ 1.7 < x \le 10.9 \\ 0.000326x^4 - 0.0185x^3 + 0.456x^2 - 5.52x + 29.9, \ 10.9 < x \le 18 \\ 0.0210x^3 - 1.39x^2 + 30.1x - 211, \ 18 < x \le 19.8 \end{cases} \quad [13]$$

where z and x denote the Cartesian coordinates of the points in the backbone curve B.

The parameterization of the boundary curve is expressed as $\mathbf{r}_\Gamma = (x, y, 0)$. The explicit expression of Γ (waterdrop) is given by

$$y = \begin{cases} 0.347x, \ 0 \le x \le 2 \\ -0.351x^7 + 7.29x^6 + 64.4x^5 + 313x^4 - 905x^3 + 1553x^2 - 1466x + 587, \ 2 < x \le 4.1 \end{cases} \quad [14]$$

where y and x are the Cartesian coordinates of the points in the boundary curve Γ.

The explicit expression of boundary curve Γ (vase) is given by

$$y = \begin{cases} 1.39, \ 0 \le x \le 0.43 \\ 0.0566x^3 + 0.376x^2 - 0.0463x + 1.40, \ 0.43 < x \le 1.7 \\ -0.000280x^4 + 0.00824x^3 - 0.129x^2 + 1.01x + 0.336, \ 1.7 < x \le 10.9 \\ 0.000195x^4 - 0.0111x^3 + 0.273x^2 - 3.31x + 17.9, \ 10.9 < x \le 18 \\ 0.0126x^3 - 0.830x^2 + 18.1x - 127, \ 18 < x \le 19.8 \end{cases} \quad [15]$$

where y and x denote the Cartesian coordinates of the points in the boundary curve Γ.

Next, the boundary curve Γ^P of the precursors is derived based on the isometry of the geodesics G and boundary curve Γ, where the parameterization of Γ^P is given by $\boldsymbol{r}_{\Gamma^P} = (x^P, y^P, 0)$. The transformation is expressed as

$$\begin{Bmatrix} x^P(s_b) \\ y^P(s_b) \end{Bmatrix} = \begin{bmatrix} \eta_x(s_b) & 0 \\ 0 & \eta_y(s_b) \end{bmatrix} \begin{Bmatrix} x(s_b) \\ y(s_b) \end{Bmatrix} \quad [16]$$

where $\eta_y(s_b) = \frac{L_G}{y} = \frac{F(\frac{\pi}{2},m)}{2E(\frac{\pi}{2},m)-F(\frac{\pi}{2},m)}$ and $\eta_x(s_b) = \frac{L'_\Gamma}{L_\Gamma}$. L_G, L_Γ ,and L'_Γ denote the half length of the geodesic curve G and the length boundary curve Γ, and the length of the boundary curve after the transformation of the y coordinates, respectively. s_b denotes the arc length coordinate along the boundary curve Γ. E and F denote the incomplete elliptic integral of the second kind and the first kind, respectively. For the axially symmetric shape, we can also assume that the boundary and backbone have the same arc length. The parameters (η_x, η_y) of the waterdrop and vase are, respectively, (0.86, 1.5) and (0.92, 2.2). Here, (η_x, η_y) does not change with s_b because we only use the information of one representative geodesic curve, which simplifies the calculation.

The boundary curve Γ^P in the waterdrop precursor is expressed as

$$y^P = \begin{cases} 0.575x^P, & 0 \le x^P \le 1.76 \\ -0.923(x^P)^7 + 16.6(x^P)^6 - 126(x^P)^5 + 529(x^P)^4 - 1314(x^P)^3 + 1939(x^P)^2 - 1569(x^P) + 538, & 1.76 < x^P \le 3.6 \end{cases} \quad [17]$$

where y^P and x^P are the Cartesian coordinates of the points in the boundary curve Γ^P.

The boundary curve Γ^P in the vase precursor is expressed as

$$y^P = \begin{cases} 3.04, & 0 \le x^P \le 0.4 \\ 0.547(x^P)^2 - 0.529x^P + 3.17, & 0.4 < x^P \le 1.59 \\ 0.00346(x^P)^3 - 0.173x(x^P)^2 + 1.88x^P + 1.22, & 1.59 < x^P \le 10.2 \\ 0.103(x^P)^2 - 2.71x^P + 23.0, & 10.2 < x^P \le 16.9 \\ -0.290(x^P)^2 + 10.7x^P - 91.2, & 16.9 < x^P \le 18.5 \end{cases} \quad [18]$$

where y^P and x^P are the Cartesian coordinates of the points in the boundary curve Γ^P.

The strain of the boundary ribbon in 2D precursors to form the target shape is in the form of $\varepsilon_r = \left[x(\max(s_b)) - x^P(\max(s_b))\right] / x^P(\max(s_b))$ and the strain to form the waterdrop and vase are 0.14 and 0.07, respectively.

Supplementary note 6. Performance of the modified kirigami grippers

Effect of the number of parallel cuts. Supplementary Fig. 13 shows the front and side views of the stretched grippers with different numbers N_C of parallel cuts at the maximum applied strain. For the grippers with small numbers N_C of cuts, as shown in Supplementary Fig. 13a ($N_C = 5$), the variation of the boundary curve (yellow curves) is constrained by the large width w_d of the discrete ribbon resulting from the small number N_C of cuts, which further, prohibits the formation and the closure of the two encapsulating hemisphere petals. With increasing number N_C of cuts and decreasing ribbon width, the boundary curves are straightened gradually (Supplementary Figs. 13b-c), with the constraint induced by the large ribbon width released. When $N_C = 37$ (Supplementary Fig. 13c), the two grasping petals become hemispheres, and the formation of the central saddle shape leads to the closure of the two hemispheres. Upon the closure of the encapsulating hemispheres, the grasping mode transfers from pinching to encapsulating, which results in a sudden jump of the pulling-out force (Supplementary Fig. 13f). It is also noteworthy that after the critical point ($N_C = 37$), increasing N_C (Supplementary Fig. 13e) barely changes the final deformed shape of the gripper, and the pulling-out force remains the same (6 N).

Supplementary note 7. Maximum tensile strain in the ribbons

The discrete ribbons buckle and suffer the maximum tensile strain ε_{max} at the top point. We demonstrate the maximum tensile strain of the representative discrete ribbon dependence on the applied strain in spheroidal, cylindrical, and saddle shapes (For the spheroidal and cylindrical shape, the representative ribbons are the discrete ribbons at the center of the morphed shape; for the saddle shape, we use the discrete ribbon at the border as the representative ribbon because there is no deformation in the central discrete ribbon before the critical strain). As shown in Supplementary Fig. 16, ε_{max} is less than 0.01 when the shapes are subject to the maximum applied strain. The maximum applied strain for the spheroidal, cylindrical, and saddle shape is 0.57, 1, and 1.53, respectively. The maximum tensile strain is given by $\varepsilon_{max} = tk_2/2$, where t is the thickness of the ribbon, k_2 is the principal curvature (Supplementary Fig. 8) at the top point of the discrete ribbon.

Supplementary note 8. Extending the strategy to other cut patterns.

Supplementary Fig. 17 shows 2D precursors composed of periodic hexagonal cuts enclosed by a circular (Supplementary Fig. 17a. i), rectangular (Supplementary Fig. 17b. i), and a biconcave (Supplementary Fig. 17c. i) boundary, respectively. Stretching the precursors leads to spheroidal (Supplementary Fig. 17a. ii), cylindrical (Supplementary Fig. 17b. ii), and saddle (Supplementary Fig. 17c. ii) shapes, respectively. First, the decreasing boundary curvature as shown in the top view (Supplementary Fig. 17a. iv) causes nonuniform expansions (rotations around the hinges relative to the neighboring units) of different unit cells leading to the formation of a spheroidal shape with positive Gaussian curvature. Second, the straight boundary (Supplementary Fig. 17b. iv) compresses the surface to induce the global out-of-plane buckling resulting in a cylinder. Third, the flattened boundary curve (Supplementary Fig. S17b. iv) leads to a saddle shape, where the local out-of-plane buckling of the unit cells at the center of the morphed shape is attributed to the geometric frustration. It is noteworthy that the local out-of-plane buckling disturbs the smoothness during the expansion of the hexagonal cuts, as shown in Supplementary Fig. 17a. iii and Supplementary Fig. 17c. iii. Also, the length of the hinge of the unit cells in the saddle shape needs to be small to reduce the bending rigidity and facilitate both the in-plane expansion and the out-of-plane buckling. These proof-of-concept results show that applying our approach to other patterns requires an additional program due to the coupling between geometric frustration and elasticity.

References

1. Love A, *A treatise on the mathematical theory of elasticity.* Cambridge, UK: Cambridge University Press (1927).

2. Timoshenko S, Gere J, *Theory of Elastic Stability.* McGraw-Hill, New York (1961).

3. Spivak M., *A Comprehensive Introduction to Differential Geometry.* (Publish or Perish, Inc., Boston), Vol I (1970).

Supplementary Figures

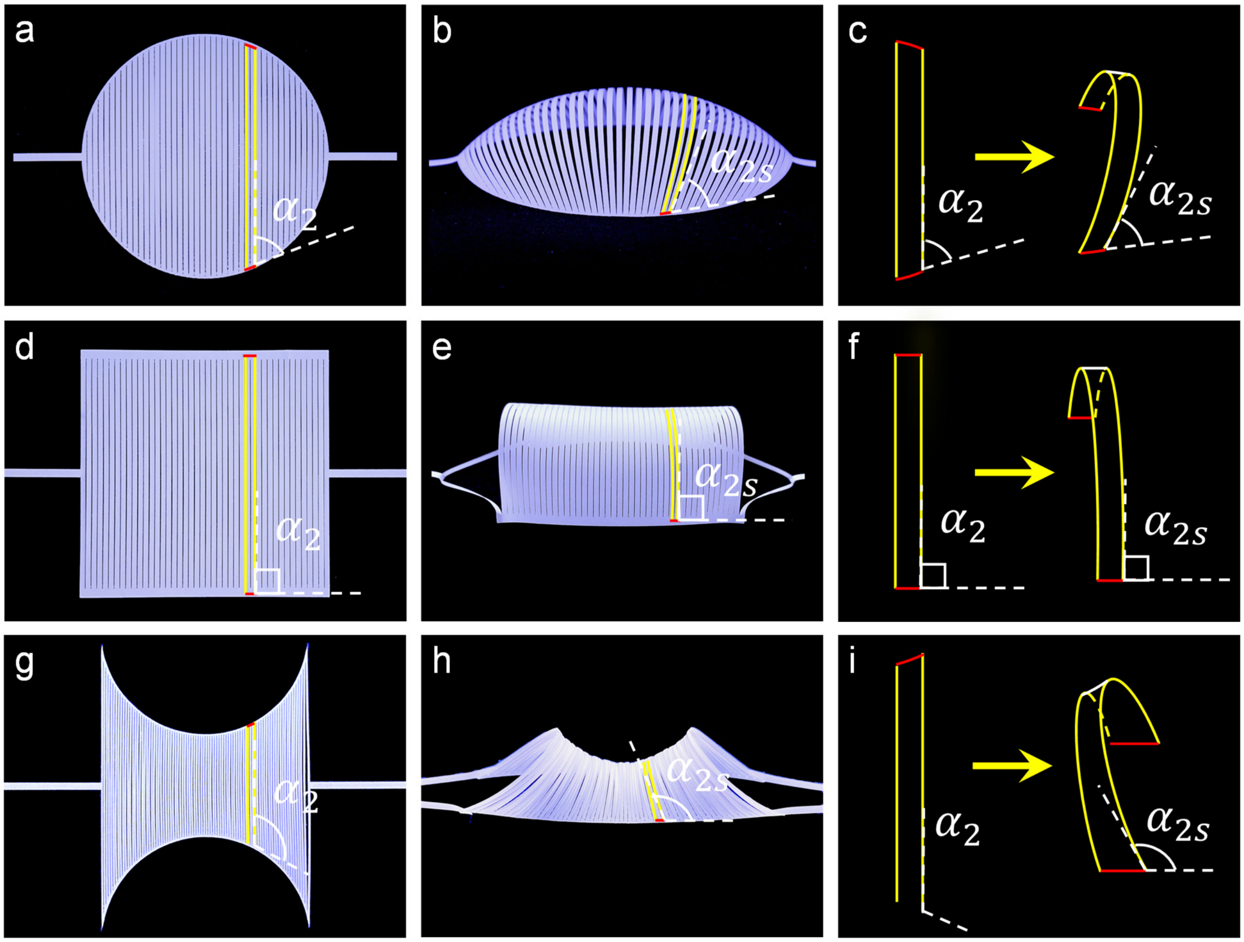

Supplementary Fig. 1. Schematics of the surface formed by neighboring discrete ribbons. **a-c** Spheroidal shape. **d-f** Cylindrical shape. **g-i** Saddle shape. **c, f, i** show, schematically, the morphed shapes formed by neighboring discrete ribbons. Red and yellow lines are boundary and discrete ribbons, respectively. α_2 and α_{2s} are the angles between the tangent line of the boundary ribbon and the tangent line of the discrete ribbon before and after the deformation, respectively.

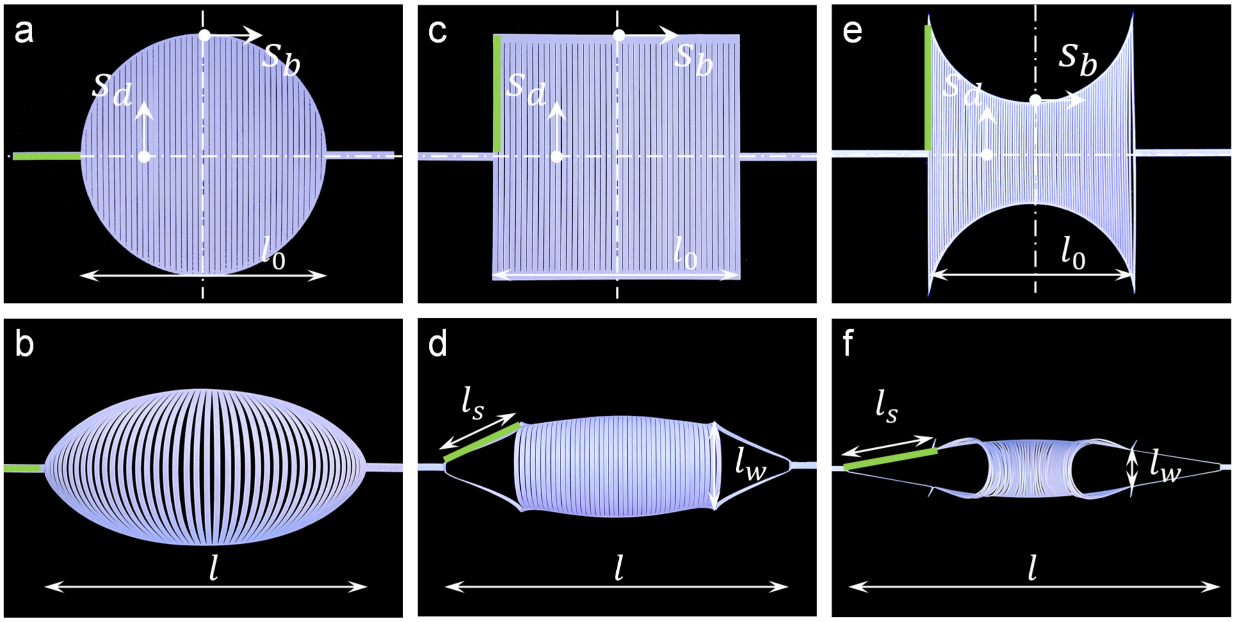

Supplementary Fig. 2. Schematics of the coordinates (top-down view). **a-b** Spheroidal shape. **c-d** Cylindrical shape. **e-f** Saddle shape. **a**, **c**, **e** are 2D precursors and **b**, **d**, **f** are morphed shapes. s_b and s_d denote the arc length coordinate of the boundary and discrete ribbons, respectively. The boundary and discrete ribbons are parametrized by arc length and the origins are located at the midpoint of ribbons. l_0 and l are the length of the shapes before and during deformation, respectively; l_s and l_w are the lengths of the stretching ribbon and the width of the shape, respectively. Green lines represent the stretching ribbons.

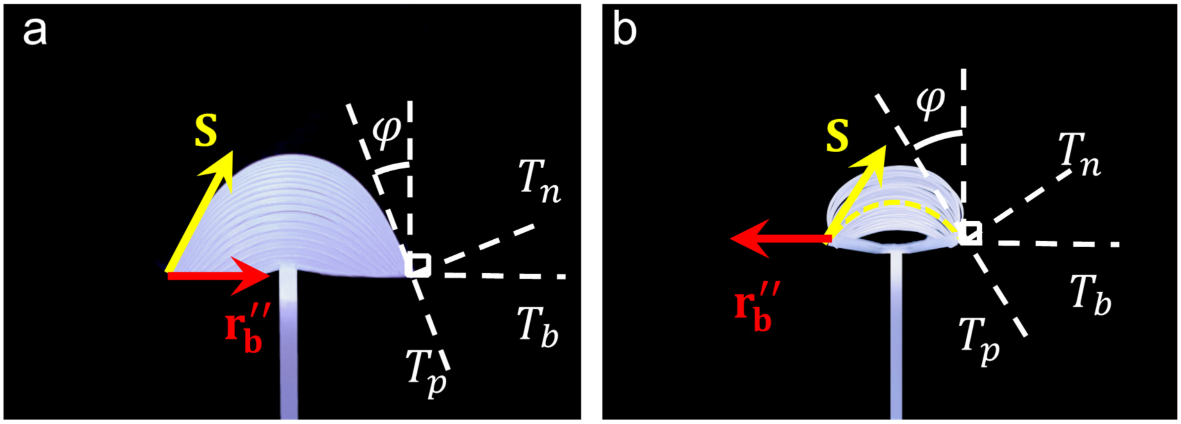

Supplementary Fig. 3. Schematics of the projection of the boundary curvature. **a** Spheroidal shape. **b** Saddle shape. The yellow vectors **S** represent the preferred normal to the boundary ribbon α_b in the surface S. The red vectors $\mathbf{r}_\mathbf{b}''$ are curvature vectors of the boundary ribbon. T_b is the plane containing the boundary curve; T_p is the tangent plane to the surface S at this point; T_n is the normal plane to the discrete ribbon at this point; φ denotes the angle between T_b and T_n.

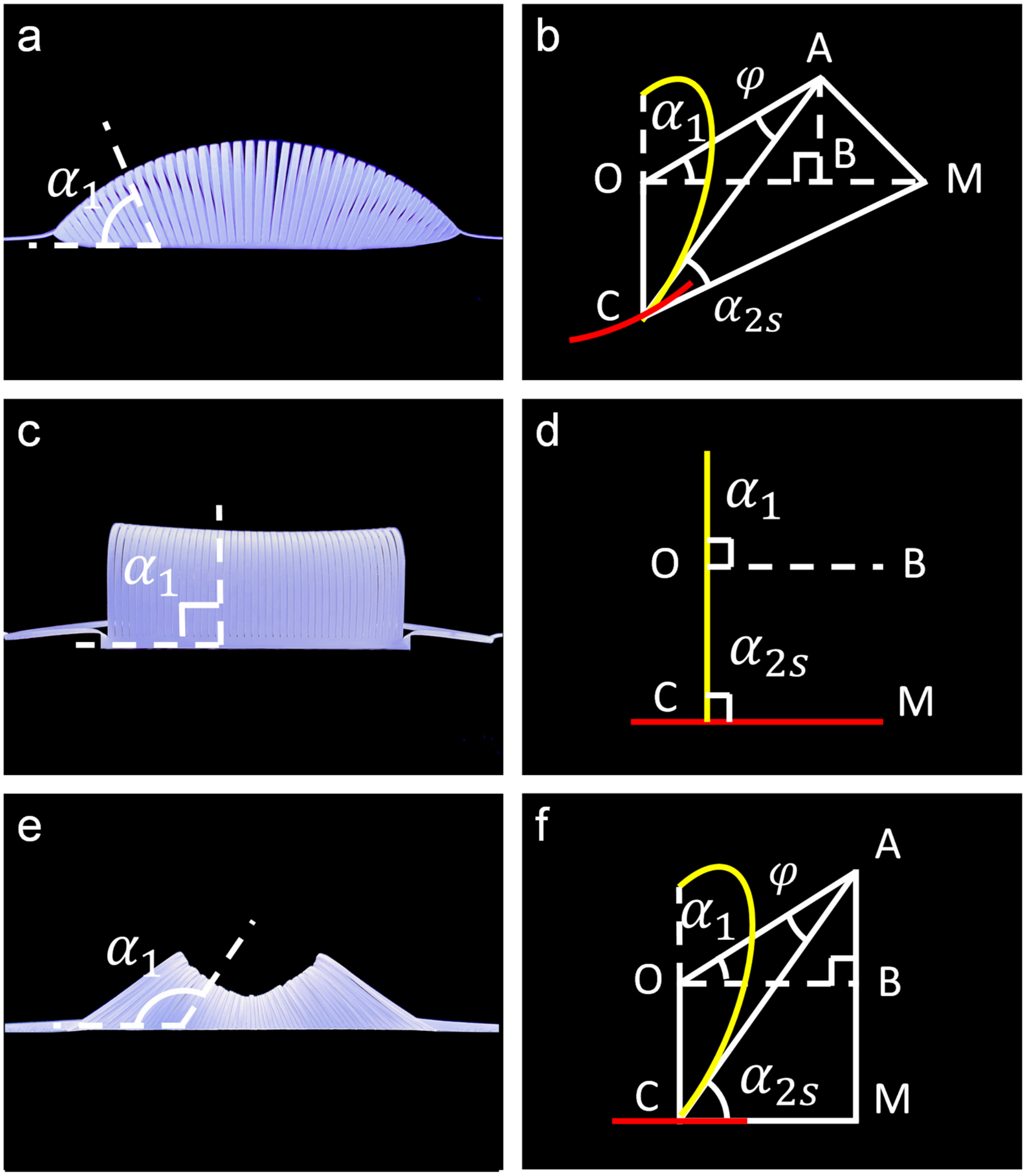

Supplementary Fig. 4. Schematics of the tilting angle of the morphed shapes. **a-b** Spheroidal shape. **c-d** Cylindrical shape. **e-f** Saddle shape. **a, c, e** The tilting angle α_1 between the plane containing the discrete ribbon and the plane containing the boundary ribbon. **b, d, f** Schematics of the relationship between angles. CM and CA are tangent to the boundary and discrete ribbons at point C, respectively; the plane $OCMB$ is the horizontal plane (the xy plane); the plane OBA is the xz plane. Red and yellow are the boundary and discrete ribbons, respectively.

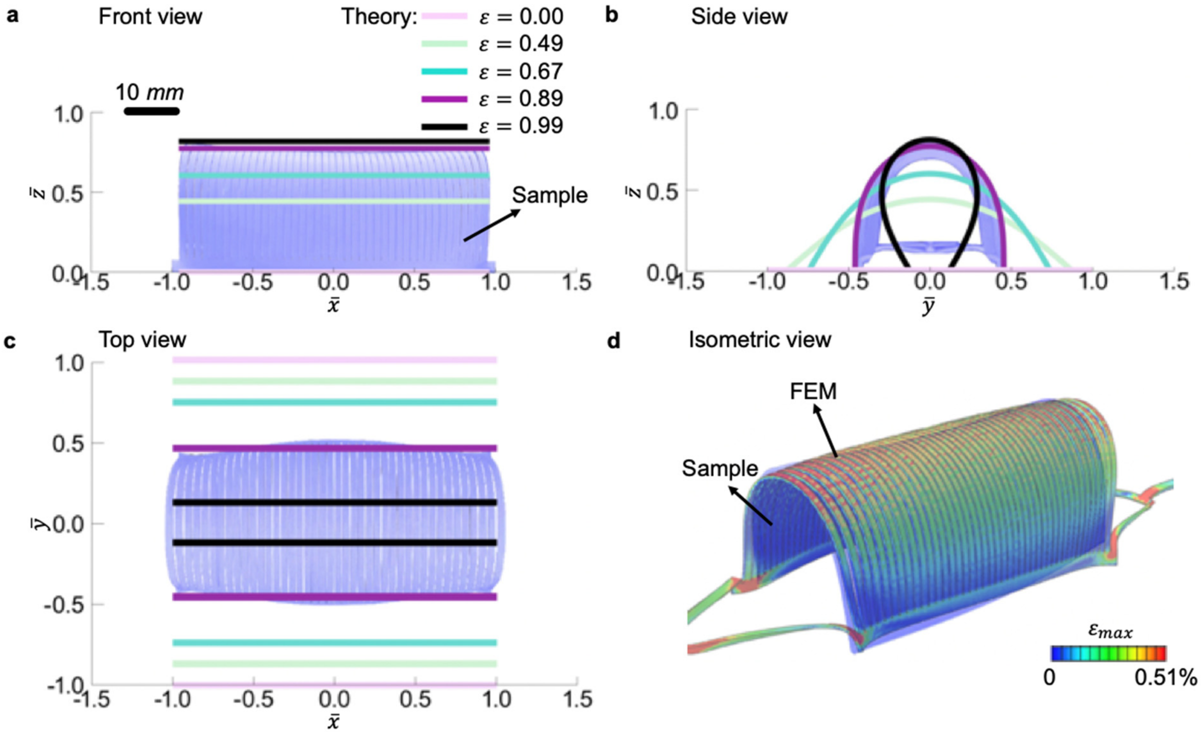

Supplementary Fig. 5. Quantifying the 3D shape shifting of the cylindrical shape through analytical modeling and simulation. **a-c** Predicted shape changes with the applied strain ε in the cylindrical sample. **a**, **b**, and **c** is the front, side, and isometric view profile, respectively. **d** is the overlapping of FEM simulation results (contours of the maximum tensile strain ε_{max}) with the experimental image at $\varepsilon = 0.89$.

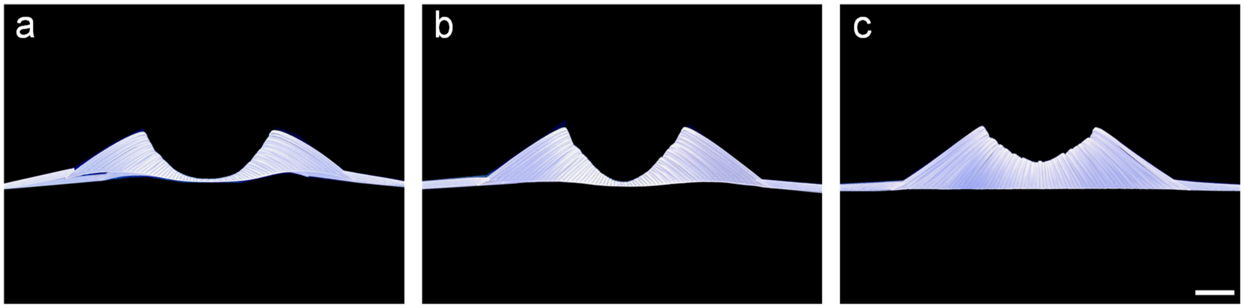

Supplementary Fig. 6. Experiments of sequential deformation in the saddle shape with increasing strain. The strains are 1.27, 1.42, and 1.47, respectively.

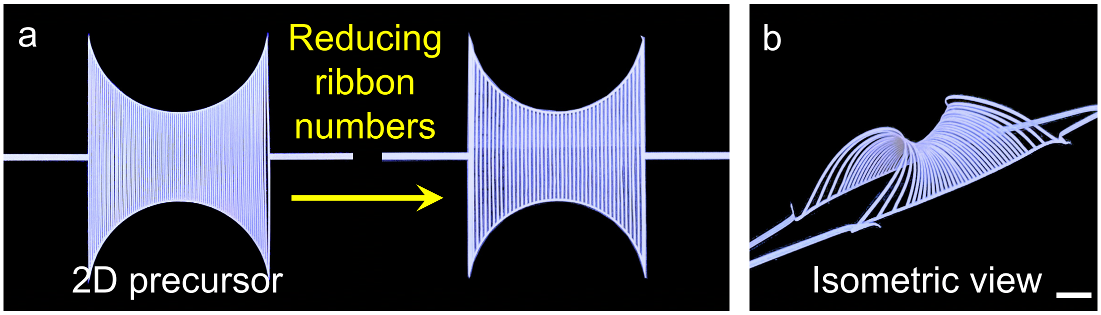

Supplementary Fig. 7. Frustration-free shape morphing in saddles. **a**. Reducing the number of parallel ribbons in the 2D biconcave precursor from 84 to 36. **b**. Isometric view of the morphed frustration-free shape without self-contacting of discrete ribbons in the center region.

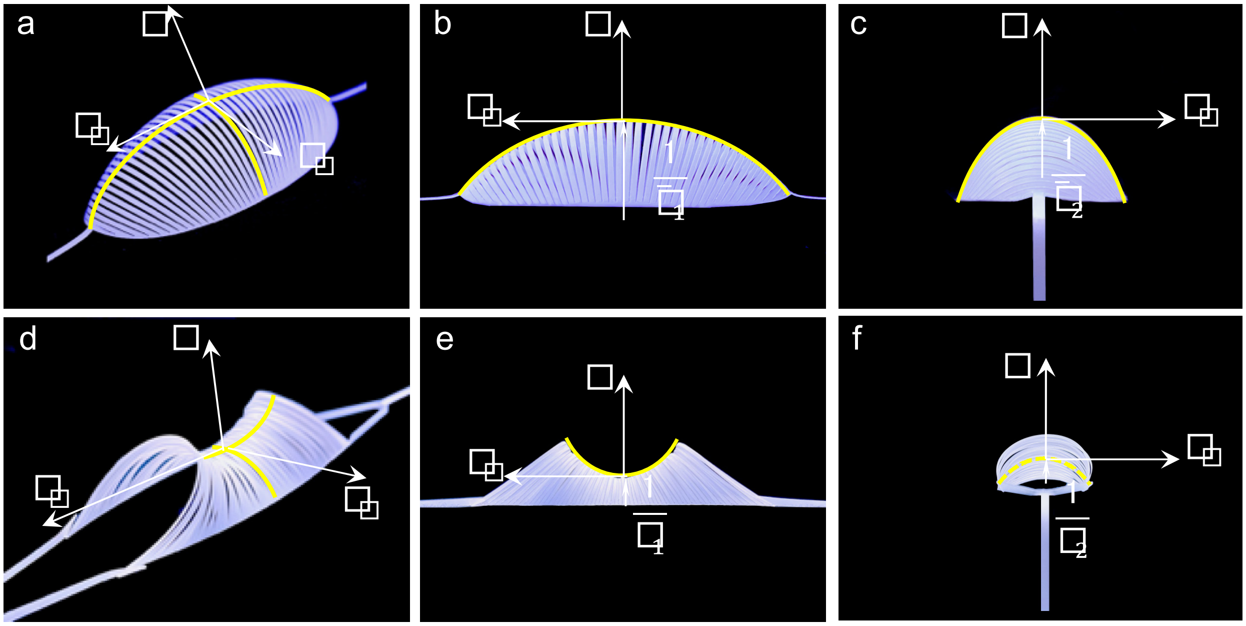

Supplementary Fig. 8. Schematics of the principal curvatures $\bar{k}_1$ and $\bar{k}_2$ at the central point. **a-c**. Spheroidal shape. **d-f** Saddle shape. **a, d** are the isometric view. **b, e** are the front view. **c, f** are the side view. **v1**, **v2**, and **N** are tangent vectors and normal vectors, respectively. Yellow lines represent the profile of the morphed shapes.

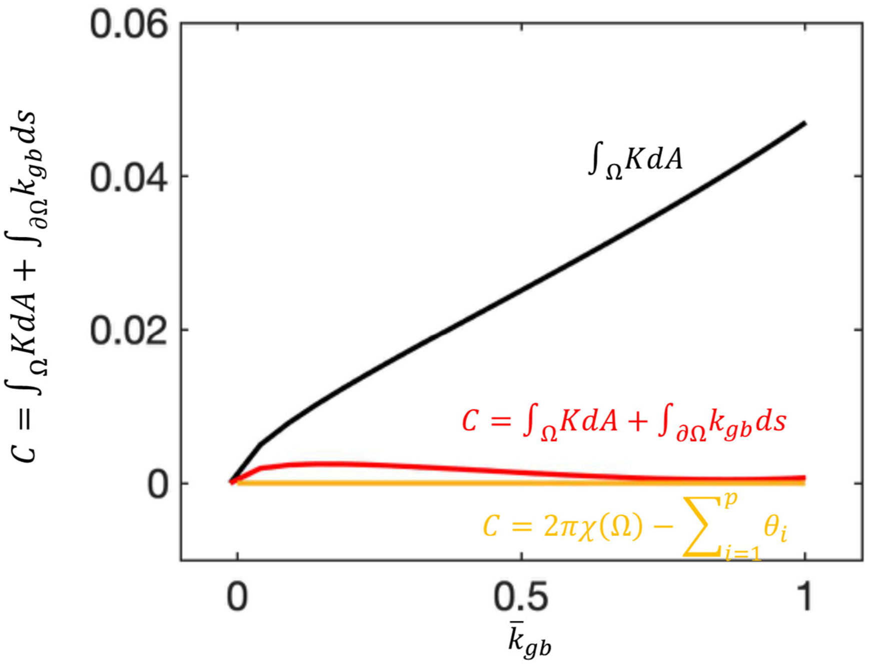

Supplementary Fig. 9. Theoretical results of C calculated from differential geometry (orange curve) and solid mechanics (red curve) model. With the black curve represents the integral of the Gaussian curvature over the surface.

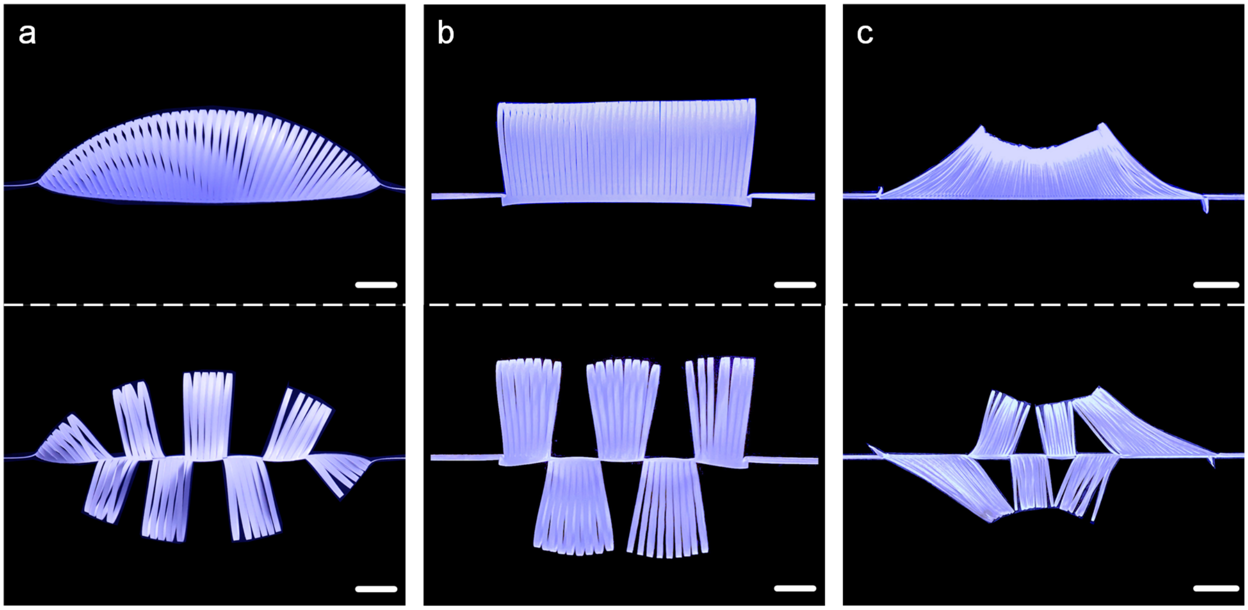

Supplementary Fig. 10. Experiments of bistable states in three characteristic structures. **a** Spheroidal shape. **b** Cylindrical shape. **c** Saddle shape. (Top) All discrete ribbons pop up. (Bottom) Some discrete ribbons pop up, with the others popping down. The applied strain is 0.35, 0.9, and 1.52, respectively. Scale bars = 10 mm.

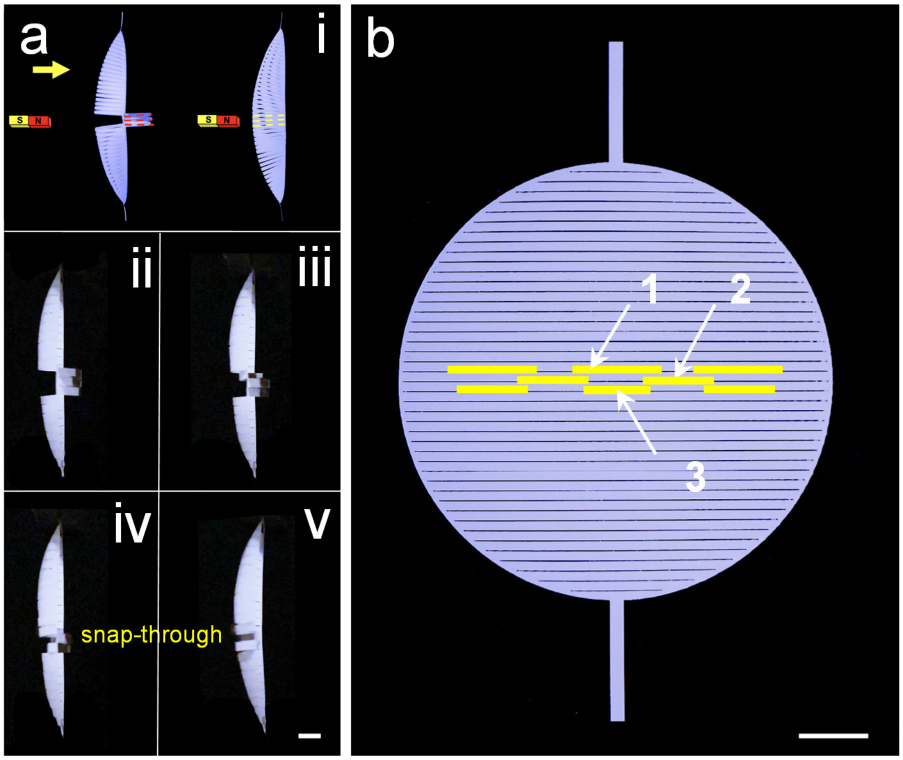

Supplementary Fig. 11. a. **ii-iv** A moving magnetic field triggers the sequential snapping of the bistable central ribbons with magnetic polymers. **b** Schematic of the distribution of the magnetic polymers attached to three discrete ribbons 1, 2, and 3. Yellow lines represent the magnetic polymers. Scale bars = 10 mm. The magnetic polymer glued to the discrete ribbons is conformable magnets for irregular surfaces (McMaster–Carr) with thickness t = 0.794 mm.

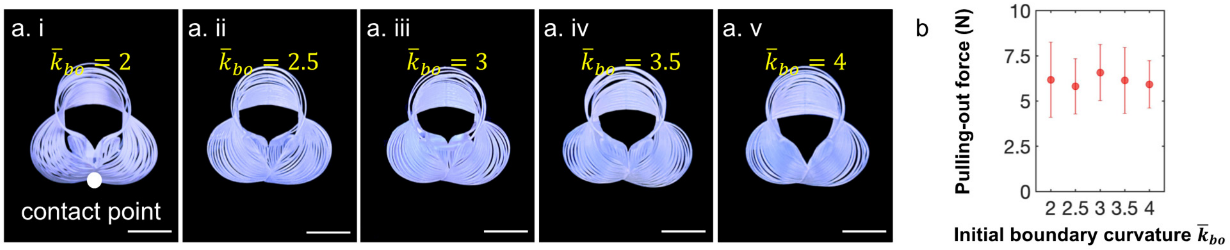

Supplementary Fig. 12. a The side view of the final deformed grippers with initial-boundary curvature $\bar{k}_{bo}$ from 2 to 4. **b** Pulling-out force test with $\bar{k}_{bo}$ from 2 to 4. The error bars represent the standard errors of the mean. Scale bars = 10 mm.

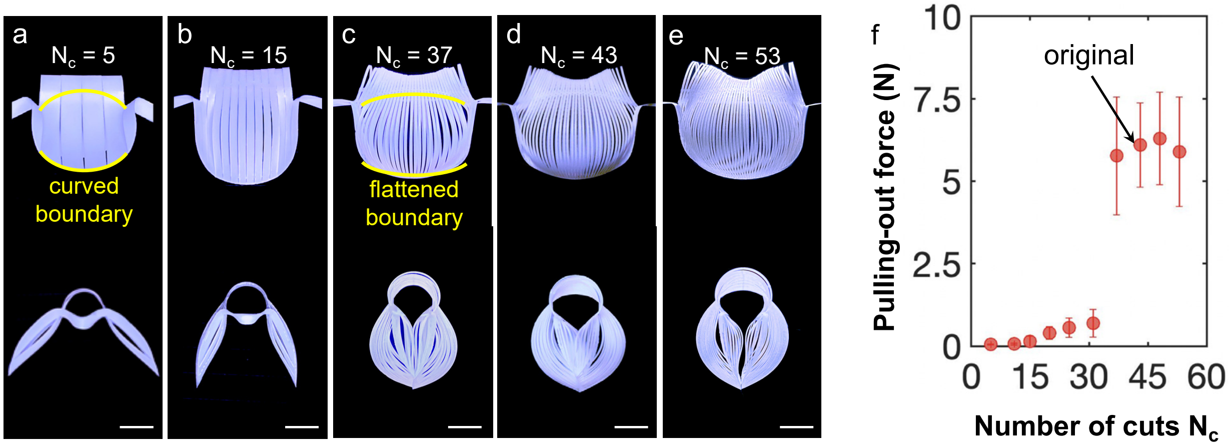

Supplementary Fig. 13. a The front and side views of the stretched grippers with different numbers N_C of parallel cuts at the maximum applied strain. The yellow curves represent the boundary curve. **b** Pulling-out force test with N_C from 0 to 60. The error bars represent the standard errors of the mean. The scale bar represents 10mm.

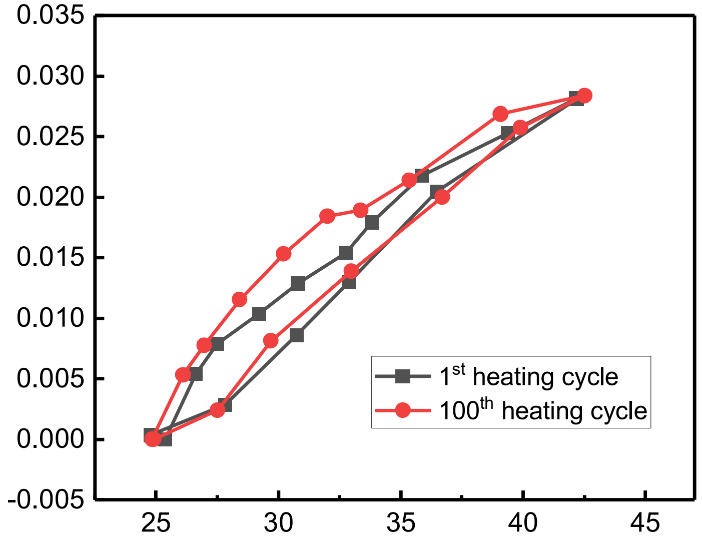

Supplementary Fig. 14. Cyclic heating-cooling test of the heater with the variation of the resistance of the heater as a function of the temperature. After 100 cycles of heating and cooling from 25°C to 42°C, the resistance-temperature curve almost does not change. The maximum difference in the resistance between the two curves induced by the heating and cooling cycles is about 0.3%.

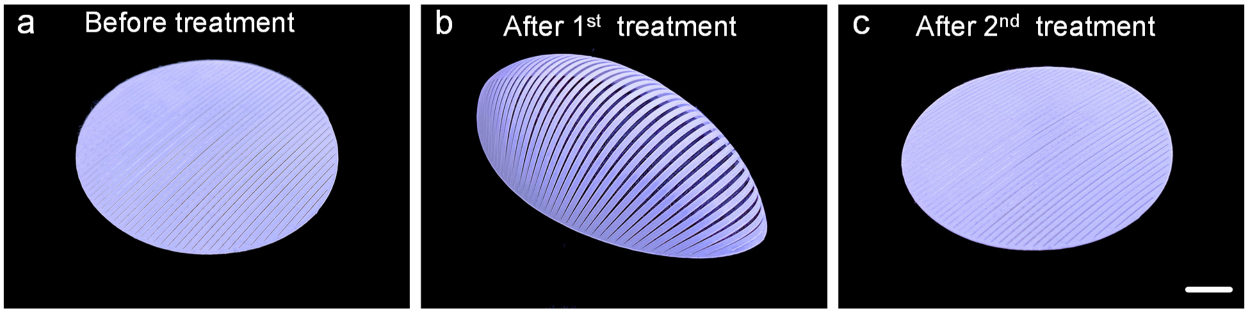

Supplementary Fig. 15. Experiments of using thermal treatment to fix the morphed shape. **a** A circular 2D precursor before the thermal treatment. **b** A spheroidal shape after the first treatment (120 ºC and 120 min). **c** Recovery to a 2D precursor after a second treatment (120 ºC and 120 min). Scale bar = 10 mm.

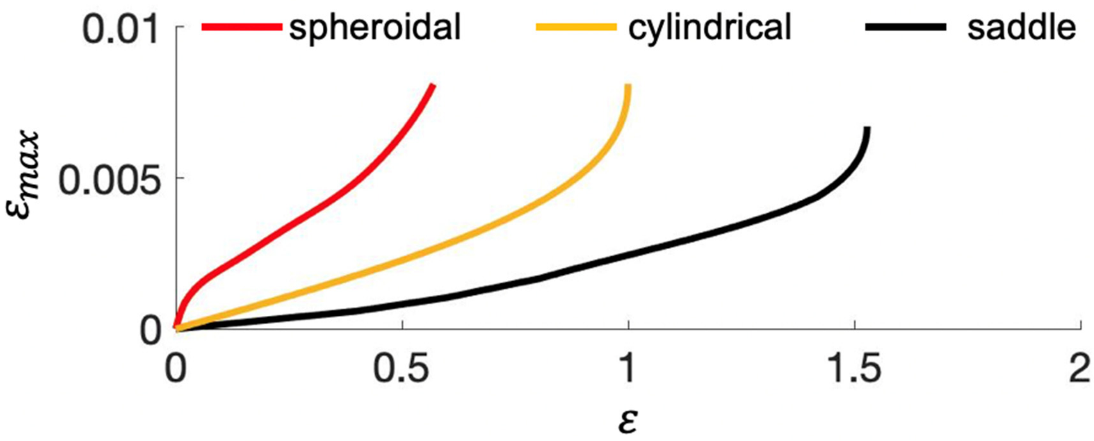

Supplementary Fig. 16. Theoretical results of the maximum tensile strain ε_{max} in the representative discrete ribbon of three characteristic shapes as a function of the applied strain ε. Red, orange, and black curves represent the spheroidal, cylindrical, and saddle shape, respectively.

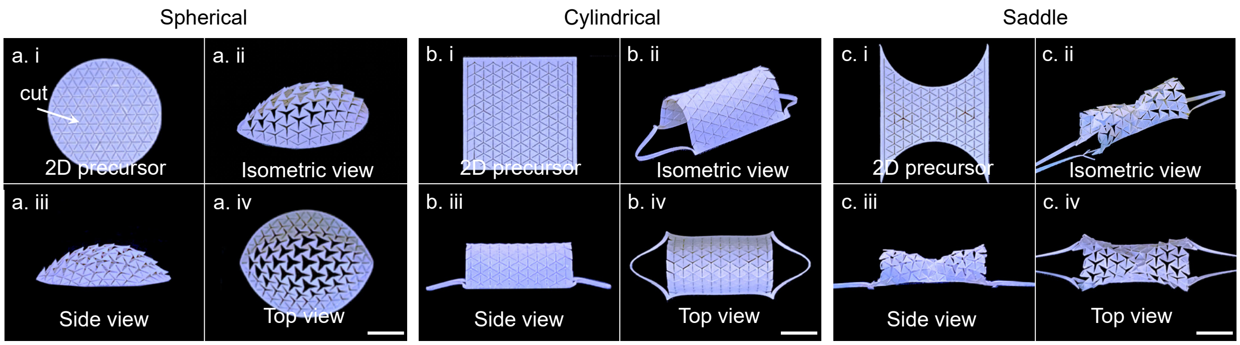

Supplementary Fig. 17. Application of our method to kirigami sheets with hexagonal patterns. **a**, **b**, and **c** are the spheroidal, cylindrical, and saddle shape formed by 2D precursors with positive, zero, and negative boundary curvature, respectively. Scale bars = 10 mm.

Legends for Supplementary Movies

Supplementary Movie 1: Demonstration of the mechanically actuated spheroidal, saddle, and cylindrical shape

Supplementary Movie 2: Demonstration of sequential snapping of the discrete ribbons in the circular precursor with attached magnetic polymers subject to a uniaxial tension (× 1/8 speed)

Supplementary Movie 3: Demonstration of the kirigami gripper grasping various objects
