# Supplementary material for: Boundary curvature guided programmable shape-morphing kirigami sheets
Source: Nat Commun. 2022 Jan 26;13:530. doi: 10.1038/s41467-022-28187-x (PMC8792031; doi:10.1038/s41467-022-28187-x)
Supplement: Supplementary file 3 — Description of Additional Supplementary Files [file 41467_2022_28187_MOESM3_ESM.pdf]

Description of Additional Supplementary Files

File Name: Supplementary Movie 1
Description: Demonstration of the mechanically actuated spheroidal, saddle, and cylindrical shape.

File Name: Supplementary Movie 2
Description: Demonstration of sequential snapping of the discrete ribbons in the circular precursor with attached magnetic polymers subject to a uniaxial tension (× 1/8 speed).

File Name: Supplementary Movie 3
Description: Demonstration of the kirigami gripper grasping various objects.
